# Supplementary material for: Precision Medicine through Next-Generation Sequencing in Inherited Eye Diseases in a Korean Cohort
Source: Genes (Basel). 2021 Dec 23;13(1):27. doi: 10.3390/genes13010027 (PMC8774510; doi:10.3390/genes13010027)
Supplement: Supplementary file 1 [file genes-13-00027-s001.zip › genes-1495226-supplementary.pdf]

Supplementary Information

Table S1. 429 target genes associated with inherited eye diseases
Table S2. Non-coding deep intronic or regulatory variants covered by the panel
Table S3. The clinical features of 90 patients who receive definite diagnosis or possible diagnosis
Table S4. The clinical features of 59 patients with unsolved cases
Table S5. The results of chromosomal comparative genomic hybridization microarray analysis
Figure S1. Pedigree of 33 patients who had family history of nystagmus
Figure S2. The distribution of age at referral for genetic testing
Figure S3. Splice site prediction analysis in 4 patients with non-canonical splice site variants.

Table S1. Target genes associated with inherited eye diseases

Targeted genes
ABCA4, ABCB6, ABHD12, ACO2, ADAM9, ADAMTS18, ADAMTSL4, ADGRV1, ADIPOR1, AFG3L2, AGBL1, AGBL5, AGK, AHI1, AIPL1, AKR1E2, ALDH18A1, ALDH1A3, ALMS1, AP3B1, ARL13B, ARL2BP, ARL3, ARL6, ASRGL1, ATF6, ATOH7, ATP1A3, ATP8A2, ATXN7, AUH, B3GLCT, BBIP1, BBS1, BBS10, BBS12, BBS2, BBS4, BBS5, BBS7, BBS9, BCOR, BEST1, BFSP1, BFSP2, BLM, BMP4, C10orf11, C12orf57, C12orf65, C19orf12, C1QTNF4, C2orf71, C5orf42, C8orf37, CA4, CABP4, CACNA1F, CACNA2D4, CAPN15, CAPN5, CASK, CBS, CC2D2A, CCDC28B, CCT2, CDH23, CDH3, CDHR1, CEP164, CEP290, CEP41, CERKL, CHD7, CHM, CHMP4B, CHN1, CHRDL1, CHST1, CHST6, CIB2, CISD2, CLK2, CLN3, CLN5, CLN6, CLN8, CLRN1, CLUAP1, CNGA1, CNGA3, CNGB1, CNGB3, CNNM4, COL11A1, COL11A2, COL17A1, COL18A1, COL25A1, COL2A1, COL4A1, COL8A2, COL9A1, COL9A2, CRB1, CRX, CRYAA, CRYAB, CRYBA1, CRYBA4, CRYBB1, CRYBB2, CRYBB3, CRYGC, CRYGD, CRYGS, CSPP1, CTNNA1, CTNS, CYP1B1, CYP27A1, CYP4V2, CYP51A1, DCDC1, DCN, DHDDS, DNAJC19, DRAM2, DTHD1, DTNBP1, EFEMP1, ELOVL4, ELP4, EMC1, EPHA2, ERCC5, ERCC6, ERCC8, EXOSC2, EYA1, EYS, FAM126A, FAM161A, FKRP, FKTN, FLVCR1, FOXC1, FOXD3, FOXE3, FOXO1, FRMD7, FSCN2, FTL, FXN, FYCO1, FZD4, GALK1, GALNS, GALT, GCNT2, GDF3, GDF6, GJA1, GJA3, GJA8, GLA, GNAT1, GNAT2, GNB3, GNPAT, GPR143, GPR179, GRK1, GRM6, GSN, GUCA1A, GUCA1B, GUCY2D, HARS, HCCS, HESX1, HGSNAT, HMCN1, HMGB3, HMX1, HOXA1, HOXB1, HSF4, IDH3B, IFT140, IMPDH1, IMPG1, IMPG2, INPP5E, INVS, IQCB1, ITM2B, JAG1, JAM3, KCNJ13, KCNV2, KIAA1549, KIF11, KIF21A, KIF7, KLHL7, KRT12, KRT3, LAMB2, LARGE1, LCA5, LCAT, LHX2, LIM2, LOXHD1, LRAT, LRIT3, LRP5, LTBP2, LZTFL1, MAB21L2, MAF, MAFB, MAK, MAPKAPK3, MC1R, MCOLN1, MERTK, MFN2, MFRP, MFSD6L, MFSD8, MIP, MIR184, MIR204, MKKS, MKS1, MTO1, MVK, MYH9, MYO7A, MYOC, NAA10, NBAS, NDP, NDUFS1, NECTIN3, NEK2, NEK8, NF2, NHS, NMNAT1, NPHP1, NPHP3, NPHP4, NR2E3, NR2F1, NRL, NYX, OAT, OCA2, OCRL, OFD1, OPA1, OPA3, OPN1LW, OPN1MW, OPN1SW, OPTN, OTX2, OVOL2, PANK2, PAX2, PAX6, PCDH15, PCYT1A, PDE6A, PDE6B, PDE6C, PDE6G, PDE6H, PDHA1, PDZD7, PEX1, PEX7, PHOX2A, PHYH, PIKFYVE, PITPNM3, PITX2, PITX3, PLA2G5, PLA2G6, POC1B, POLG, POMGNT1, PPT1, PRCD, PRDM13, PRDM5, PRKAA2, PROM1, PRPF3, PRPF31, PRPF4, PRPF6, PRPF8, PRPH2, PRSS56, PXDN, RAB28, RAB3GAP1, RAB3GAP2, RARB, RAX, RAX2, RBP3, RBP4, RCBTB1, RD3, RDH12, RDH5, RECQL4, RGR, RGS9, RGS9BP, RHO, RIMS1, RLBP1, RNLS, ROBO3, ROM1, RP1, RP1L1, RP2, RP9, RPE65, RPGR, RPGRIP1, RPGRIP1L, RS1, RTN1IP1, SAG, SALL4, SDCCAG8, SEMA4A, SH3PXD2B, SHH, SIL1, SIX3, SIX6, SLC16A12, SLC24A1, SLC24A5, SLC25A46, SLC33A1, SLC38A8, SLC45A2, SLC4A11, SLC7A14, SNRNP200, SNX3, SOX2, SOX3, SPATA7, SPG7, SRD5A3, SREBF2, STRA6, TACSTD2, TAT, TCF4, TCTN1, TCTN2, TCTN3, TDRD7, TEAD1, TENM3, TFAP2A, TGFBI, TIMM8A, TIMP3, TMEM114, TMEM126A, TMEM138, TMEM216, TMEM231, TMEM237, TMEM67, TMEM70, TMEM98, TOPORS, TP63, TPP1, TRIM32, TRNT1, TRPM1, TSPAN12, TTC21B, TTC8, TTLL5, TUB, TUBB3, TULP1, TYR, TYRP1, UBUAD1, UCHL1, UNC119, USH1C, USH1G, USH2A, VAX1, VCAN, VIM, VPS13B, VSX1, VSX2, WDPCP, WDR19, WFS1, WHRN, WT1, YMEL1L1, ZEB1, ZNF408, ZNF423, ZNF469, ZNF513

Table S1: Genes included in inherited eye diseases target enrichment. Listed are the genes included in the custom designed target enrichment along with the disease or phenotype associated with the gene according to Online Medelian Inheritance in Man (OMIM), OMIM phenotype identification number, and OMIM or Gene Cards gene identification number. Genes are named according HUGO Gene Nomenclature Committee (HUGO, http://www.genenames.org) approved nomenclature.

Table S2. Non-coding deep intronic or regulatory variants covered by the panel

Gene HGNC	Genomic location hg19	HGVS	Refseq
ABCA4	Chr1:94526934	c.1938-619A>G	NM_000350.2
ABCA4	Chr1:94525509	c.2160+584A>G	NM_000350.2
ABCA4	Chr1:94576926	c.302+68C>T	NM_000350.2
ABCA4	Chr1:94509799	c.3050+370C>T	NM_000350.2
ABCA4	Chr1:94493272	c.4539+1729G>T	NM_000350.2
ABCA4	Chr1:94484082	c.5196+1056A>G	NM_000350.2
ABCA4	Chr1:94484001	c.5196+1137G>A	NM_000350.2
ABCA4	Chr1:94484001	c.5196+1137G>T	NM_000350.2
ABCA4	Chr1:94566773	c.570+1798A>G	NM_000350.2
ABCA4	Chr1:94468019	c.6148-471C>T	NM_000350.2
ATP1A3	Chr19: 42470891	c.*196_*198dupCTC	NM_001256213.1
BBS1	Chr11:66291105	c.951+58C>T	NM_024649.4
BBS4	Chr15:73001821	c.77-220delA	NM_033028.4
BBS5	Chr2:170354110	c.619-27T>G	NM_152384.2
C5orf42	Chr5: 37157484	c.7957+288G>A	NM_023073.3
CEP290	Chr12:88494960	c.2991+1655A>G	NM_025114.3
CEP290	Chr12:88462434	c.6012-12T>A	NM_025114.3
CHM	ChrX:85223644	c.315-4587T>A	NM_000390.2
COL11A1	Chr1:103488576	c.1027-24A>G	NM_080629.2
COL11A1	Chr1:103386637	c.3744+437T>G	NM_080629.2
COL11A1	Chr1:103491958	c.781-450T>G	NM_080629.2
COL2A1	Chr12:48379984	c.1527+135G>A	NM_001844.4
CTNS	Chr17: 3552117	c.141-24T>C	NM_001031681.2
DHDDS	Chr1:26774026	c.441-24A>G	NM_024887.3
ELP4	Chr11:31685945	c.1143+14176C>A	NM_019040.4
ERCC6	Chr10: 50681659	c.2599-26A>G	NM_000124.3
ERCC8	Chr5: 60223645	c.173+1046A>G	NM_001290285.1
FKTN	Chr9: 108368857	c.648-1243G>T	NM_001079802.1
FOXC1	Chr6:1610252	c.-429C>G	NM_001453.2
FOXC1	Chr6:1610437	c.-244C>T	NM_001453.2
FOCX1	Chr6:1613076	c.*734A>T	NM_001453.2
FRMD7	ChrX:131228285	c.285-118C>T	NM_194277.2
FTL	Chr19: 49468583	c.[-178T>G;-182C>T]	NM_000146.3

FTL	Chr19: 49468587	c.-168G>A	NM_000146.3
FTL	Chr19: 49468601	c.-164C>A	NM_000146.3
FTL	Chr19: 49468604	c.-161C>T	NM_000146.3
FTL	Chr19: 49468605	c.-160A>G	NM_000146.3
FTL	Chr19: 49468606	c.-159G>C	NM_000146.3
FTL	Chr19: 49468616	c.-149G>C	NM_000146.3
GALT	Chr9: 34646583	c.-67-52_-67-49del	NM_000155.3
GALT	Chr9: 34648519	c.687+66T>A	NM_000155.3
GALT	Chr9: 34649617	c.1059+56C>T	NM_000155.3
GLA	ChrX: 100654735	c.639+919G>A	NM_000169.2
GLA	ChrX: 100654793	c.640-859C>T	NM_000169.2
GNAT2	Chr1:110151229	c.461+24G>A	NM_005272.3
GPR143	ChrX:9711844	c.659-131T>G	NM_000273.2
GPR143	ChrX:9708630	c.885+748G>A	NM_000273.2
IMPDH1	Chr7: 128043703	c.402+57G>A	NM_000883.3
LCAT	Chr16: 67976512	c.524-22T>C	NM_000229.1
MYO7A	Chr11:76893448	c.3109-21G>A	NM_000260.3
NF2	Chr22: 30050946	c.516+232G>A	NM_000268.3
NMNAT1	Chr1:10003561	c.-69C>T	NM_022787.3
NMNAT1	Chr1:10003560	c.-70A>T	NM_022787.3
OAT	Chr10: 126100239	c.199+303C>G	NM_000274.3
OCRL	ChrX:128687279	c.239-4023A>G	NM_000276.3
OFD1	ChrX:13773245	c.1130-22_1130-19delAATT	NM_003611.2
OFD1	ChrX:13768358	c.935+706A>G	NM_003611.2
OPN1MW	ChrX: 153448055	c.-112A>C	NM_000513.2
OVOL2	Chr20:18038552	c.-274T>G	NM_021220.2
OVOL2	Chr20:18038585	c.-307T>C	NM_021220.2
OVOL2	Chr20:18038648	c.-370T>C	NM_021220.2
PAX6	Chr11:31816377	c.524-41T>G	NM_000280.4
PDHA1	ChrX: 19372579	c.511-30G>A	NM_000284.3
PDHA1	ChrX: 19373648	c.759+26G>A	NM_000284.3
PDHA1	ChrX: 19377850	c.*79_*90dupAGTCAATGAAAT	NM_000284.3
PEX7	Chr6: 137143759	c.-45C>T	NM_000288.3
PITX2	Chr4:111538827	c.*454C>T	NM_000325.5
PPT1	Chr1: 40539204	c.*526_*529delATCA	NM_000310.3

PROM1	Chr4: 15985995	c.2281-26_2281-17del	NM_006017.2
PROM1	Chr4:15989860	c.2077-521A>G	NM_006017.2
PRPF31	Chr19:54633399	c.1374+654C>G	NM_015629.3
SDCCAG8	Chr1: 243468435	c.740+356C>T	NM_001350246.1
TIMM8A	ChrX:100601671	c.133-23A>C	NM_004085.3
TRNT1	Chr3: 3188088	c.609-26T>C	NM_001302946.1
USH2A	Chr1:216247476	c.5573-834A>G	NM_206933.2
USH2A	Chr1:216064540	c.7595-2144A>G	NM_206933.2
USH2A	Chr1:216039721	c.8845+628C>T	NM_206933.2
USH2A	Chr1:215967783	c.9959-4159A>G	NM_206933.2
WFS1	Chr4: 6295693	c.713-1075C>G	NM_001145853.1

Table S3. The clinical features of 90 patients who receive definite diagnosis

Patient No.	Initial Clinical Impression	Molecular diagnosis	Diagnosis After revisit	Sex	Age (y)	Nystagmus	Refraction RE	Refraction LE	BCVA	Fundus	ERG	Additional phenotype
1	Cone-rod dystrophy	*ABCA4*	Cone-rod dystrophy	F	74.9	No	-0.325	-0.5	FC/FC	Macular atrophy with pigmentary changes	Extinguished	None
2	LCA	*AHI1*	Joubert syndrome	M	24.3	2Hz pendular	0.25	0	0.2/0.3	Pigmentary retinopathy	Extinguished	Delayed development
3	Laurence-Moon syndrome	*BBS1*	Bardet-Biedl syndrome	F	23.8	5-6Hz pendular	-2.5	-3.25	0.05/0.05	Granular pigmentation on macula	Extinguished	Obesity Polydactyly hyperlipidemia
4	IIN	*CACNA1F*	CSNB	M	1.2	1Hz LBJ	3.25	3.25	CSM/CSM	Normal	Inconclusive	None
5	LCA	*CACNA1F*	CSNB	M	1.8	2Hz pendular	-2	-2	UCSM/UCSM	Normal	Inconclusive	None
6	IIN	*CACNA1F*	CSNB	M	4.3	2Hz pendular	0.5	0.5	0.4/0.4	Normal	Inconclusive	None
		LRP5	r/o FEVR									
7	IIN	*CACNA1F*	CSNB	M	4.3	2Hz RBJ-LBJ	1.25	2	0.1/0.5	Normal	Negative ERG	None
8	IIN	*CACNA1F*	CSNB	M	1.3	4Hz pendular	-4.5	-3	CSM/CSM	Mild tessellated	Negative ERG	None
9	LCA	*CEP290*	Joubert syndrome	F	0.5	Wandering movement	7	6	UCSM/ UCSM	Normal	Extinguished	MRI: molar tooth, Delayed development
10	Achromatopsia	*CNGA3*	Achromatopsia	M	8.3	4Hz pendular	1.25	0.375	HM/HM	Grossly normal	Extinguished in photopic	None
11	Stickler syndrome	*COL2A1*	Stickler syndrome	M	0.8	No	-10.5	-10.5	CSM/CSM	Vitreous strand at periphery	NA	Hypotonia, Abnormal skull shape
12	Pierre Robin sequence	*COL2A1*	Stickler syndrome	F	10.9	No	-9	-10	0.6/0.5	Vitreous strand at periphery	NA	Cleft palate Pierre Robin
13	LCA	*CRX*	LCA	M	30.7	Wandering movement	5.5	6.5	HM/HM	Macular atrophy & Pigmentary retinopathy	Extinguished	None
14	Congenital cataract	*CRYGC*	Congenital cataract	F	1	Multidirectional nystagmus	18.5	18.5	UCSM/UCSM	Normal	NA	None
15	RP	*EYS*	RP	M	23.1	No	-1.25	-1	0.7/0.8	Pigmentary retinopathy	Extinguished in scotopic	None
16	IIN	*FRMD7*	IIN	M	1	3Hz pendular	1	-0.125	CSM/CSM	Normal	NA	None
17	IIN	*FRMD7*	IIN	M	16.3	2Hz LBJ	-6	-7	0.6/0.6	Normal	NA	None
18	IIN	*FRMD7*	IIN	F	5.3	2Hz LBJ	0.25	0.25	0.2/0.2	Normal	NA	None
19	IIN	*FRMD7*	IIN	M	6.5	2Hz pendular	-0.75	-0.5	0.6/0.6	Normal	NA	None
20	IIN	*FRMD7*	IIN	M	0.5	1Hz pendular	1	0	CSM/CSM	Normal	NA	None
21	IIN	*FRMD7*	IIN	F	9.2	2HZ LBJ	-1.5	-1	1.0/1.0	Normal	NA	None

22	IIN	*FRMD7*	IIN	M	14.3	2Hz pendular	-5.25	-6.5	0.8/1.0	Normal	NA	None
23	IIN	*FRMD7*	IIN	F	34.8	4Hz LBJ	-8	-8	0.7/0.8	Normal	NA	None
24	IIN	*FRMD7*	IIN	F	15.8	2Hz LBJ	-2	-2.5	1.0/1.0	Normal	NA	None
25	IIN	*FRMD7*	IIN	M	10.1	2Hz pendular	0.5	0.25	1.0/1.0	Normal	NA	None
26	IIN	*FRMD7*	IIN	M	3.3	2Hz pendular	2.75	2.75	CSM/CSM	Normal	NA	None
27	IIN	*FRMD7*	IIN	M	1.8	2Hz pendular	0.5	0.5	CSM/CSM	Normal	NA	None
28	IIN	*FRMD7*	IIN	M	30.3	2Hz pendular	-1	-1.5	0.9/1.0	Normal	NA	None
29	IIN	*FRMD7*	IIN	M	4.3	2Hz LBJ	plano	plano	0.8/1.0	Normal	NA	None
30	IIN	*FRMD7*	IIN	M	11.2	2Hz RBJ-LBJ	3	3	1.0/1.0	Normal	NA	None
31	IIN	*FRMD7*	IIN	M	11.5	2Hz LBJ	-1	-1	1.0/0.9	Normal	NA	None
32	IIN	*FRMD7*	IIN	M	22.3	3Hz RBJ	-2.5	-4.75	0.8/0.8	Normal	NA	None
33	IIN	*FRMD7*	IIN	M	2	3Hz pendular	-1	-1.5	0.9/1	Normal	NA	None
34	IIN	*FRMD7*	IIN	M	8.3	3Hz pendular	-1.25	-0.75	0.7/0.7	Normal	NA	None
35	IIN	*FRMD7*	IIN	M	10.1	3Hz pendular	0.75	1	0.3/0.3	Normal	NA	None
36	IIN	*FRMD7*	IIN	M	5.3	3Hz pendular	plano	plano	0.8/0.8	Normal	NA	None
37	IIN	*FRMD7*	IIN	M	20.1	3Hz LBJ	-8.75	-3.75	1/1	Normal	NA	None
38	Congenital cataract	*FTL*	Hyperferritinemia -cataract syndrome	M	7.8	No	-0.5	-0.5	0.6/0.4	Normal	NA	High ferritin level
39	FEVR	*FZD4*	FEVR	F	8.3	No	-2.25	-6	1.0/0.7	Retinal temporal dragging	NA	None
40	FEVR	*FZD4*	FEVR	M	24.3	3Hz pendular	-6.25	-4	0.2/0.2	Retinal temporal dragging	NA	None
41	FEVR	*FZD4*	FEVR	M	3.2	No	-10.5	-10.75	CSM/CSM	Retinal temporal dragging	NA	None
42	Congenital cataract	*GJA3*<br>*OPA1*	Congenital cataract<br>Dominant optic atrophy	M	14.6	Multidirectional nystagmus	9.25	13.0	0.3/0.1	Optic atrophy	NA	None
43	Ocular albinism	*GPR143*	Ocular albinism	M	33.1	2Hz pendular	-4	-4.25	0.15/0.2	Depigmented fundi	NA	None
44	Ocular albinism	*GPR143*	Ocular albinism	M	10.3	3Hz RBJ	2.75	2.75	0.05/0.05	Depigmented fundi	NA	None
45	Ocular albinism	*GPR143*	Ocular albinism	M	20.6	2-3Hz pendular	-5	-4.75	0.2/0.3	Depigmented fundi	NA	None
46	Ocular albinism	*GPR143*	Ocular albinism	M	1.3	2Hz pendular	1.5	1.5	CSM/CSM	Depigmented fundi	NA	None
47	Ocular albinism	*Xp22.3 deletion*	Multiple syndrome including ocular albinism	M	5.1	2Hz LBJ pendular	1.5	1.5	UCSM/UCSM	Depigmented fundi	NA	Ichyosis, Delayed development, Hypothyroidism
48	Ocular albinism	*GPR143*	Ocular albinism	M	8.9	3Hz pendular	1.25	4	0.15/0.15	Depigmented fundi	NA	None

49	LCA	*GUCY2D*	LCA	F	0.6	2Hz vertical pendular	4.75	4.75	UCSM/UCSM	Normal	Extinguished	None
50	LCA	*GUCY2D*	LCA	M	0.4	Wandering movement	7	7	UCSM/UCSM	Normal	Extinguished	None
51	LCA	*GUCY2D*	LCA	F	0.6	Wandering movement	4	3.75	UCSM/UCSM	Normal	Extinguished	None
52	IIN	*GUCY2D*	LCA	F	1.9	3Hz LBJ	-1	-0.75	CSM/CSM	Normal	Extinguished	None
53	CCDD	*KIF21A*	CCDD	F	12.5	No	-0.75	-0.25	1.0/0.5	Normal	NA	None
54	FEVR	*LRP5*	FEVR	F	4.8	No	0	-16	1/0.04	Retinal temporal dragging	NA	None
55	Congenital cataract	*NHS*	Nance-Horan syndrome	F	5.4	No	-0.75	0.5	0.3/0.5	Normal	NA	Atrial septal defect<br>Febrile seizure
56	LCA	*NMNAT1*	LCA	F	1.4	Wandering movement	5	5.5	UCSM/UCSM	Macular atrophy & Pigmentary retinopathy	Extinguished	None
57	LCA	*NMNAT1*	LCA	M	0.5	Wandering movement	4.75	7.25	UCSM/UCSM	Macular atrophy & Pigmentary retinopathy	Extinguished	None
58	Optic atrophy	*NR2F1*	BBSOAS	M	19.1	Latent nystagmus	0.75	1.25	0.9/0.2	Optic atrophy	NA	Intellectual disability, Delayed development
59	Optic atrophy	*NR2F1*	BBSOAS	M	6.6	Latent nystagmus	-0.25	+0.25	0.2/0.2	Optic atrophy	Inconclusive ERG	Intellectual disability, Delayed development
60	IIN	*NYX*	CSNB	M	1.2	4Hz pendular	-3.5	-3	CSM/CSM	Normal	Negative ERG	None
61	Unexplained visual loss	*NYX*	CSNB	M	5.2	No	-6	-6.5	0.4/0.4	Tesellated fundus	Negative ERG	None
62	Optic atrophy	*OPA1*	Dominant optic atrophy	M	6.5	3-4Hz multidirectional	1	1.5	0.02/0.05	Optic atrophy	NA	None
63	Optic atrophy	*OPA1*	Dominant optic atrophy	M	9	No	0	-1	0.4/0.5	Optic atrophy	NA	None
64	PAX6-related phenotype	*PAX6*	PAX6-related phenotype	M	19.6	Dysconjugate 3Hz LBJ	-10.5	-10	0.5/0.5	Foveal hypoplasia	Normal	None
65	PAX6-related phenotype[a]	*PAX6*	PAX6-related phenotype	F	5.7	1-2Hz pendular	-3	-3	0.2/0.3	Foveal hypoplasia	NA	None
66	PAX6-related phenotype	*PAX6*	PAX6-related phenotype	F	7.9	No	-6	-5.5	0.15/0.15	Foveal hypoplasia	NA	None
67	PAX6-related phenotype	*PAX6*	PAX6-related phenotype	M	5.6	Gaze-evoked nystagmus	-4.75	-3	0.1/0.1	Foveal hypoplasia	NA	None

68	PAX6-related phenotype	*PAX6*	PAX6-related phenotype	M	0.4	2Hz multidirectional -UBJ	-1.5	-1.5	UCSM/UCSM	Foveal hypoplasia	NA	None
69	PAX6-related phenotype	*PAX6*	PAX6-related phenotype	M	0.4	3Hz vertical c multidirectional nystagmus	7	7	UCSM/UCSM	Foveal hypoplasia	NA	None
		OPN1LW	*Color blindness protan*									
70	PAX6-related phenotype	*PAX6*	PAX6-related phenotype	F	6.2	3Hz multidirectional	2	2	0.15/0.15	Foveal hypoplasia	NA	None
71	Achromatopsia	*PDE6C*	Achromatopsia	M	5.3	2Hz pendular	-6	-6	0.1/0.05	Pigmentary retinopathy	Extinguished in photopic	None
72	IIN	*PDE6C*	Achromatopsia	M	4.3	3Hz pendular	2.25	3	0.10/0.15	Normal	Extinguished in photopic	None
73	Optic atrophy	*PDHA1*	Leigh syndrome	M	3.8	No	-0.25	-0.25	CSM/CSM	Optic atrophy	NA	Delayed development
74	RP	*PRPH2*	Cone dystrophy	M	7.6	No	-4.5	-7	0.9/0.8	Normal	Severe attenuated	None
75	CSNB	*RHO*	CSNB	M	27.9	No	-7	-10	0.05/0.05	Tesellated fundus	Negative ERG	None
76	Cone dystrophy	*RP1*	Retinitis pigmentosa	M	19.8	No	-7.5	-7.5	0.6/0.6	Pigmentary retinopathy	Severe attenuated	None
77	LCA	*RPGRIP1*	LCA	F	4.3	Infrequent UBJ	3	2.5	0.2/0.2	Pigmentary retinopathy	Extinguished	None
78	PAX6-related phenotype	*SLC38A8*	SLC38A8 associated foveal hypoplasia	M	15.8	2-3Hz bidirectional jerk	0.5	0.7	0.2/0.3	Foveal hypoplasia	NA	None
79	Ocular albinism	*SLC38A8*	SLC38A8 associated foveal hypoplasia	M	0.6	2Hz bidirectional jerk	0.25	1.5	CSM/CSM	Foveal hypoplasia Depigmented fundi	NA	None
80	PAX6-related phenotype	*SLC38A8*	SLC38A8 associated foveal hypoplasia	M	10.4	3Hz bidirectional jerk	1	1.5	0.5/0.3	Foveal hypoplasia	NA	None
81	Oculocutaneous albinism	*SLC45A2*	Oculocutaneous albinism	M	8.9	2Hz pendular	-5	-4.5	0.5/0.5	Depigmented fundi	NA	Brownish hair
82	LCA	*SPATA7*	LCA	F	7.2	Wandering movement	8	9.5	LP/LP	Pigmentary retinopathy	Extinguished	None
83	LCA	*TUBB3*	Cortical dysplasia	M	0.6	Wandering movememtn	1.5	2.0	UCSM/UCSM	Normal	NA	Delayed development
84	Oculocutaneous albinism	*TYR*	Oculocutaneous albinism	F	26.3	2-3Hz pendular	1.25	2.75	0.8/0.6	Depigmented fundi	NA	None
85	Usher syndrome	*USH2A*	Usher syndrome	M	52.8	No	-4.5	-5.25	0.3/0.2	Pigmentary retinopathy	Extinguished	Sensorineural hearing loss

86	Usher syndrome	*USH2A*	Usher syndrome	F	33	No	-4.75	-3.75	1.0/0.9	Pigmentary retinopathy	Severe attenuated in scotopic	Sensorineural hearing loss
87	X-linked retinoschisis	*VPS13B*	Cohen syndrome	M	5.3	No	-4	-3.5	0.1/0.1	Cystoid retinoschisis Pigmentary retinopathy	Severe attenuated	Microcephaly Delayed speech Slender finger Delayed development
88	Corneal dystrophy	*ZEB1*	PPMD	M	11.3	No	-15	-13	0.3/0.2	Normal	NA	None
89	Corneal dystrophy	*ZEB1*	PPMD	F	4.3	No	-9	-7.5	0.7/0.8	Normal	NA	None
90	Optic atrophy	*12p12 deletion*	SOX5-associated optic atrophy	F	8.1	No	-2	-2.25	0.6/0.4	Optic atrophy	NA	Dysmorphic face Development delay hypertrichosis

ADHD attention deficit hyperactivity disorder, *BBSOAS* Bosch Boonstra Schaaf optic atrophy syndrome, *BCVA* best corrected visual acuity, *CCDD* congenital cranial dysinnervational disorder, *CSM* constant, steady, and maintained fixation, *CSNB* congenital stationary night blindness, *F* female, *CF* count finger, *FEVR* familial exudative vitreoretinopathy, *HM* had motion, *IIN* idiopathic infantile nystagmus, *LBJ* left beat jerk, *LCA* Leber congenital amaurosis, *LE* left eye, *LP* light perception, *M* male, *NA* not available, PPMD posterior polymorphous corneal dystrophy, *RBJ* right beat jerk, *RE* right eye, *RP* retinitis pigmentosa, *UCSM* no constant, no steady, no maintained fixation, *UBJ* upbeat nystagmus

[a]This patient with *PAX6* variant exhibited normal iris structure.

Table S4. The clinical features of 59 unsolved patients

Patient No.	Initial Clinical Impression	Sex	Age (y)	Nystagmus	Refraction		BCVA	Fundus	ERG	Additional phenotype
					RE	LE				
91	IIN	M	7.5	2Hz pendular	0.5	0.5	0.4/0.4	Normal	NA	None
92	IIN	M	5.9	2Hz pendular	-1.5	0.75	0.2/0.15	Normal	Diminished photopic and scotopic	None
93	RP	F	63.9	No	-0.5	-0.5	1/0.7	Pigmentary retinopathy	Diminished photopic and scotopic	None
94	LCA	M	0.5	Wandering eye movement	5	5	UCSM/UCSM	Marbled fundus	Extinguished	None
95	RP	M	63.1	No	-0.12	0.75	0.5/0.6	Pigmentary retinopathy	Decreased scotopic	None
96	Oculocutaneous albinism	M	9.1	3Hz pendular	0.25	0.75	0.2/0.2	Depigmented fundi	NA	Brownish hair
97	IIN	F	8.2	3Hz LBJ	-2.25	-4.5	0.2//0.2	Retinal vessel straightening	NA	None
98	RP	F	34.9	No	-10	-10.5	0.1/0.15	Pigmentary retinopathy	Extinguished in photopic	Delayed development
99	FEVR	F	25.3	6Hz oblique rotatory	-7	-7	0.1/0.08	Retinal temporal dragging	NA	None
100	Optic atrophy	M	5.3	No	1.25	1.5	0.5/0.2	Optic atrophy	NA	Development delay, cerebellar atrophy
101	IIN	F	5.8	2Hz upbeat nystagmus	0	0.5	0.8/0.8	Normal	NA	None
102	Congenital cataract	F	14.5	No	1.75	-1.25	0.8/1.0	Normal	NA	None
103	Optic atrophy	M	8.6	No	0	-0.25	0.5/0.7	Optic atrophy	NA	None
104	Unexplained visual loss	F	7.8	No	-0.5	-0.5	0.05/0.1	Normal	Normal	None
105	Unexplained visual loss	F	6.4	No	0	0.5	0.5/0.3	Normal	Normal	None
106	IIN	F	5.6	2Hz pendular	-4	-5	0.1/0.05	Normal	Inconclusive ERG	None
107	IIN	F	30	5Hz LBJ	-8.5	-8	0.5/0.5	Normal	Negative ERG	None
108	Optic atrophy	F	44.2	2Hz LBJ	-5.75	-8	0.1/0.1	Optic atrophy	NA	Muscle cramps
109	IIN	M	27.6	3Hz RBJ	-3.75	-2.5	0.9/1	Normal	NA	None
110	IIN	M	19.6	multidirectional	-6.25	-6.5	0.1/0.1	Normal	Normal	None
111	IIN	F	6	multidirectional	-2	-1.75	0.2/0.3	Normal	Inconclusive ERG	None
112	IIN	M	9.5	3Hz LBJ	0.5	0	1.0/1.0	Normal	NA	None
113	IIN	M	44.8	3Hz RBJ	-0.5	-1	1.0/1.0	Normal	NA	None
114	Congenital cataract	M	15.3	Multidirectional	11	-14	0.5/0.02	Normal	NA	None
115	IIN	M	16.3	2Hz LBJ	-9	-15	0.1/0.2	Myopic fundus	NA	Developmental delay
116	IIN	M	20.8	3Hz RBJ-LBJ	-8	-5.5	0.7/0.6	Normal	NA	None
117	Peter's anomaly	M	2.1	multidirectional	-2	NA	UCSM/ UCSM	Not clearly visualized	NA	None
118	IIN	F	4.7	3Hz pendular	-1.75	-2.25	0.3/0.2	Normal	NA	None

119	FEVR	F	11.5	3Hz LBJ	0.75	1.5	0.05/0.02	Retinal temporal dragging	NA	None
120	IIN	M	10.7	2Hz LBJ	-3.75	-2	0.30.5	Normal	NA	None
121	Optic atrophy	M	15.9	Manifest latent	-7	-6	0.5/0.1	Optic atrophy	Normal	None
122	Ocular albinism	M	3	Gaze-evoked	-6	-7	0.3/0.3	Depigmented fundi	Normal	None
123	IIN	F	15.8	3Hz RBJ	0	-0.5	1.0/1.0	Normal	Normal	None
124	RP	M	20.7	No	-8	-8.5	0.5/0.5	Pigmentary retinopathy	Diminished scotopic	None
125	Optic atrophy	M	9.4	Spasmus-nutans like nystagmus	1	1	0.08/0.3	Optic atrophy	NA	None
126	RP	M	76.4	No	-1.5	-1.5	HM/0.5	Pigmentary retinopathy	Diminished scotopic, photopic	None
127	IIN	M	1.3	3Hz pendular	-2	-2	CSM/CSM	Normal	NA	None
128	Optic atrophy	F	8.2	No	2.25	2.25	0.5/0.5	Optic atrophy	NA	None
129	Optic atrophy	F	14.8	No	-4.75	-3.5	0.15/0.5	Optic atrophy	NA	None
130	IIN	M	7.4	2Hz pendular	-0.75	-1.25	0.5/0.7	Normal	NA	None
131	Optic atrophy	M	19	No	-5.5	-6.25	0.02/1.0	Optic atrophy	NA	None
132	Optic atrophy	F	4	Latent nystagmus	-0.25	-0.25	0.2/0.3	Optic atrophy	NA	None
133	Optic atrophy	M	13.3	Latent nystagmus	-6.0	-3.5	0.3/0.5	Optic atrophy	NA	Delayed development Intellectual disability
134	IIN	M	2.1	2Hz RBJ-LBJ	plano	plano	CSM/CSM	Normal	NA	None
135	Macular dystrophy	F	78	No	+1.25	+0.75	0.5/0.2	Macular dystrophy	Severely attenuated	None
136	RP	F	30.6	No	plano	plano	1.0/0.8	Pigmentary retinopathy	Diminished scotopic	None
137	IIN	M	1.2	3Hz pendular	-0.25	-0.25	CSM/CSM	Normal	NA	None
138	Optic nerve hypoplasia	M	42.3	Multidirectional nystagmus	-2.75	-2.75	HM/0.6	Optic nerve hypoplasia	NA	None
139	RP	F	66.8	No	-1.5	0.25	0.02/0.2	Pigmentary retinopathy	Severely attenuated	None
140	IIN	F	4.3	3Hz RBJ-LBJ	0.125	0.125	0.5/0.5	Normal	NA	None
141	IIN	M	9.2	3Hz RBJ	-0.25	-0.50	1.0/0.8	Normal	NA	None
142	IIN	M	2.1	3Hz RBJ-LBJ	-0.5	-0.5	CSM/CSM	Normal	NA	None
143	IIN	M	12.1	3Hz pendular	-1	-2.25	0.9/0.9	Normal	NA	None
144	Occult macular dystrophy	M	60.7	No	-3.25	0	0.9/1.0	Normal	Multifocal ERG: diminished response in fovea	None
145	Optic atrophy	F	12.3	No	plano	-0.25	1.0/0.6	Optic atrophy	NA	None
146	Optic atrophy	M	4.8	No	0.125	plano	0.5/0.5	Optic atrophy	NA	None
147	Optic atrophy	M	9.9	No	-3.25	-3.75	0.4/0.2	Optic atrophy	NA	None
148	Optic atrophy	M	61.9	No	-0.5	-0.5	0.5/0.9	Optic atrophy	NA	None
149	Rod-cone dystrophy	F	15.3	No	-1	-1.5	0.15/0.5	Paravenous pigmentary retinopathy	Diminished scotopic	None

BCVA best corrected visual acuity, *CSM* constant, steady, and maintained fixation, *ERG* electroretinogram, *F* female, *FEVR* familial exudative vitreoretinopathy, *HM* hand motion, *IIN* idiopathic infantile nystagmus, *LBJ* left beating jerk, *LE* left eye, *M* male, *NA* not available, *RBJ* right beat jerk, *RE* right eye, *RP* retinitis pigmentosa, *UCSM* no constant, steady, or maintained fixation.

Table S5. The Results of chromosomal comparative genomic hybridization microarray analysis

Patient No.	Copy number analysis by targeted NGS	Chromosomal microarray analysis	Deleted genes	Phenotypes	Inheritance	MIM	Ophthalmic features	Reported systemic features
47	Xp22.3 micro deletion	Xp22.33p.22.2 (3489126_10217107)	*GPR143*	Ocular albinism, type I	XL	300808	Nystagmus, Depigmented fundi Chiasmal misrouting	None
			CLCN4	Mental retardation, X-linked	XLR	300114	None	Microcephaly, delayed psychomotor development, hypotonia
			KAL1	Kallmann syndrome 1	XLR	300836	None	Hypogonadism, anosmia Decreased fertility, delayed puberty
			NLGN4	Mental retardation, X-linked	XLR	300427	None	Autism, Mental retardation
			STS	Itchyosis, X-linked	XLR	300747	None	Brownish scales
90	12p12 micro deletion	12p12.2p12.1 (20286266_25154015)	*ABCC9*	Cantu syndrome	AD	601439	Epicanthal folds Long eyelashes	Congenital hypertrichosis, neonatal macrosomia, cardiomegaly, pericardial effusion
			GYS2	Glycogen storage disease 0, liver	AR	138571	None	Diabetes, hypoglycemia, ketonuria arrhythmia
			LDHB	Lactate dehydrogenase-B deficiency	AR?	150100	None	Coronary artery disease?
			PYROXD1	Myopathy, myofibrillar, 8	AR	617220	None	Limb weakness, myopathy
			SLCO1B1	Hyperbilirubinemia, Rotor type, digenic	DR	604843	None	Hyperbilirubinemia
			SLCO1B3	Hyperbilirubinemia, Rotor type, digenic	DR	605495	None	Hyperbilirubinemia
			SOX5	Lamb-Shaffer syndrome	AD	604975	Non-progressive optic atrophy, exotropia, and moderate myopia	Developmental delay, speech delay, hypotonia, strabismus, dysmorphic features

AD autosomal dominant, *AR* autosomal recessive, *DR* digenic recessive, *XL* X-linked, *XLR* X-linked recessive

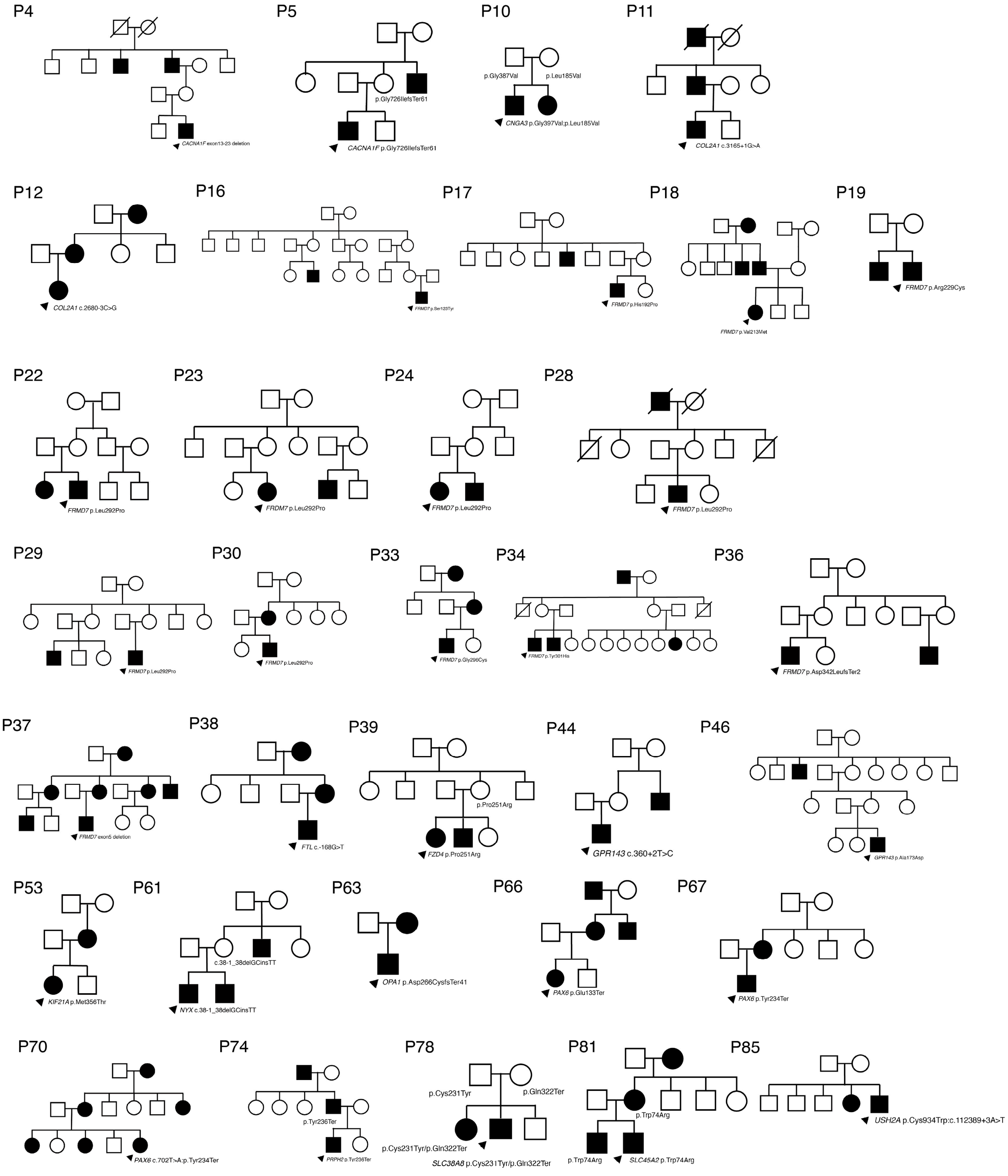

Figure S1. Pedigree of 33 patients who had family history with definite diagnosis after targeted next-generation sequencing

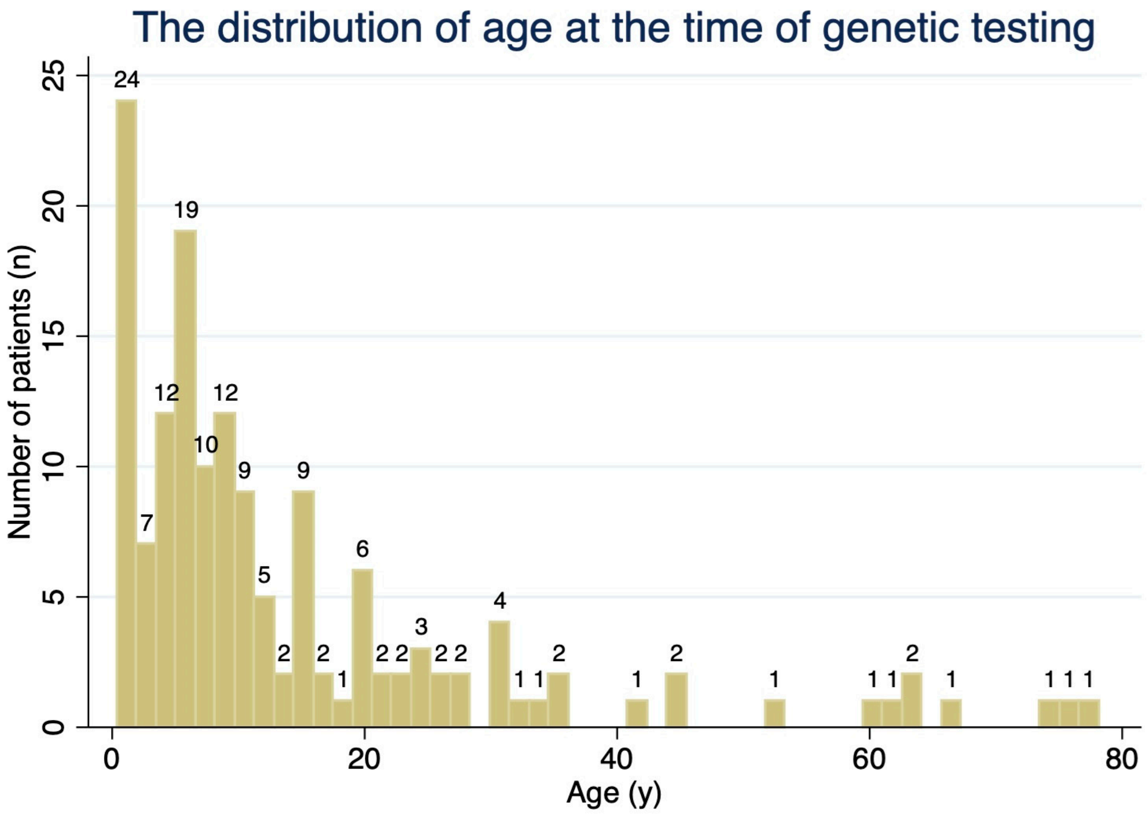

Figure S2. The distribution of age at the time of referral for genetic testing

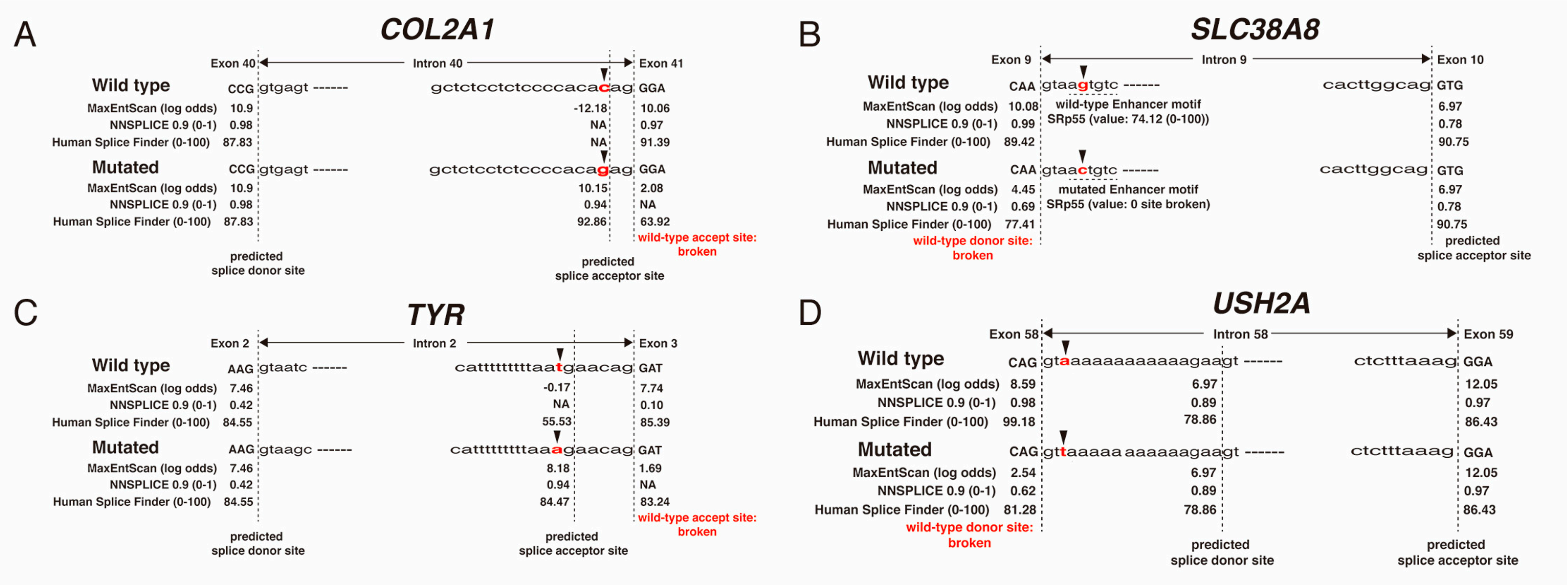

Figure S3. Splice site prediction analysis in 4 patients with non-canonical splice site variants. Arrowheads indicate the mutated base. The scores calculated using MaxEntScan, NNSPLICE, and Human Splicing Funder v3.0 are depicted above each splice site. A higher score predicts a strong splice site. The values provided in parentheses indicate the score ranges in each algorithm.

(A) (P12) Predictions of splice sites in the wildtype and mutated *COL2A1*. Diagram of the *COL2A1* region comprising exons 40 and 41. The *COL2A1* c.2680-3C>G variant predicted to create new splice acceptor site. SpliceAI predicted the loss of a splice acceptor site 3bp downstream (delta: 0.95) and the gain of a splice acceptor site 1bp downstream (delta score: 0.99). (B) (P79) Predictions of splice sites in the wildtype and mutated *SLC38A8*. Diagram of the *SLC38A8* region comprising exons 8 and 9. The *SLC38A8* c.1214+5G>C variant predicted to cause the loss of enhancer SRp55 motif. SpliceAI predicted the loss of a splice donor site 5bp upstream (delta: 0.96). (C) (P84) Predictions of splice sites in the wildtype and mutated *TYR*. Diagram of the *TYR* region comprising exons 2 and 3. The *TYR* c.1037-7T>A variant predicted to create new

splice acceptor site. SpliceAI predicted the loss of a splice acceptor site 7bp downstream (delta: 0.29) and the gain of a splice acceptor site 2bp downstream (delta score: 0.95). (D) (P85) Predictions of splice sites in the wildtype and mutated *USH2A*. Diagram of the *USH2A* region comprising exons 58 and 59. The *USH2A* c.11389+3A>T variant predicted to create new splice donor site. SpliceAI predicted the loss of a splice donor site 3bp upstream (delta: 0.90).
